# Clinical efficacy and safety of atezolizumab plus bevacizumab versus lenvatinib in the treatment of advanced hepatocellular carcinoma: A systematic review and meta-analysis

**DOI:** 10.1097/MD.0000000000033852

**Published:** 2023-06-09

**Authors:** Sihao Du, Ke Cao, Zhenshun Wang, Dongdong Lin

**Affiliations:** a Department of General Surgery, Xuanwu Hospital, Capital Medical University, Beijing, China; b Department of General Surgery, Beijing Youan Hospital, Capital Medical University, Beijing, China; c Beijing Institute of Hepatology, Beijing Youan Hospital, Capital Medical University, Beijing, China.

**Keywords:** atezolizumab, bevacizumab, hepatocellular carcinoma, lenvatinib

## Abstract

Assess the effectiveness and safety of treatment options atezolizumab plus bevacizumab (Atez/Bev) or lenvatinib in clinical practice for patients with advanced hepatocellular carcinoma (HCC) patients.

**Methods:**

To compare the effectiveness of Atez/Bev and lenvatinib in treating advanced HCC, we systematically searched the PubMed, EMBASE, and Web of Science databases. We utilized Review Manager 5.3 to extract and analyze the data.

**Results:**

The present systematic review included 8 nonrandomized studies comprising a total of 6628 cases. There was no significant difference in 0.5-, 1-, 1.5-year OS rates and 0.5-, 1-year PFS rates between the 2 groups. However, patients with HCC caused by viral hepatitis would benefit more from the Atez/Bev therapy (hazard ratio = 0.75, 95% confidence interval: 0.63–0.89) but patients with a Child–Pugh class B liver function would benefit more from lenvatinib (hazard ratio = 1.70, 95% confidence interval: 1.07–2.70). At the same time, there are no major differences in safety between the 2 treatment options.

**Conclusion:**

Our study did not find any significant difference in effectiveness and safety between Atez/Bev and lenvatinib. However, Additional verification is required to determine whether these 2 therapeutic approaches have varying effects on distinct populations.

## 1. Introduction

Globally, primary liver cancer has the sixth morbidity in all cancer types, the second morbidity of the digestive tract tumor and caused the fourth leading number of cancer-related mortality.^[1]^ Among all histological types, hepatocellular carcinoma (HCC) has the highest incidence rate and often developed in the background of hepatitis B virus (HBV) infection, most patients have HBV hepatitis and liver cirrhosis at the same time.^[2]^ For early-stage liver cancer, radical treatments like surgical resection, local ablation, and liver transplantation are suitable options for a cure. In fact, the median survival period for such treatments can exceed 5 years.^[3,4]^ Regrettably, the majority of HCC patients are diagnosed in the intermediate or advanced stages.^[5]^ In such cases, surgical resection is usually not the recommended course of treatment and patients should opt for non-surgical local treatments and systemic therapy instead. Unfortunately, the median survival period for these patients is still less than 2 years because of the high rate of recurrence and metastasis.^[3,4,6]^

At present, there are 3 regimens available for first-line treatment: Sorafenib, lenvatinib and atezolizumab plus bevacizumab (Atez/Bev). According to the REFLECT study, the lenvatinib arm exhibited a comparable overall survival (OS) to the Sorafenib arm (with a median of 13.6 and 12.3 months, respectively), and further showed significantly enhanced progression-free survival (PFS, 7.4 months vs 3.7 months; *P* < .001) and objective response rate (ORR, 24.1% vs 9.2%; *P* < .001).^[7]^ At the same time, the IMbrave 150 study revealed that Atez/Bev provided a benefit in terms of OS (19.2 months vs 13.4 months; *P* < .001) and PFS (6.9 months vs 4.3 months; *P* < .001) when compared to Sorafenib. The ORR for the Atez/Bev arm was 30%, which was significantly higher than the 11.9% observed in the Sorafenib arm (*P* < .001).^[8]^ Both lenvatinib and Atez/Bev have demonstrated superiority over Sorafenib, however, there is still controversy over which one is more suitable as the first-line treatment option. Some studies suggest that Atez/Bev is more effective than lenvatinib in prolonging patient survival,^[9–11]^ while others have reached the opposite conclusion,^[12,13]^ and some people believe that there is no difference in the efficacy of the 2 treatments.^[14–16]^

We performed a comprehensive review of relevant literature in this study to assess the clinical efficacy and safety of Atez/Bev versus lenvatinib for the treatment of HCC. We hope our study can provide clinicians with accurate information to guide their decision-making process.

## 2. Materials and methods

As a systematic review and meta-analysis, this study does not necessitate a declaration of Institutional Review Board or similar formal research ethics committee approval, including the corresponding decision/protocol number. Nonetheless, we obtained a PROSPERO (Registered) ID for the study, which is CRD42023404298, http://links.lww.com/MD/J119.

### 2.1. Literature search strategy

The literature search procedure entailed performing an extensive search across PubMed, Embase, and Web of Science. The scope of the search covered the time frame from the earliest available date until February 2023. In PubMed, the search was executed using a combination of keywords and MeSh terms, specifically “Atezolizumab,” “Bevacizumab,” “lenvatinib,” and “Hepatocellular carcinoma,” http://links.lww.com/MD/J118.

### 2.2. Study selection

Selection of studies: The process for determining which studies to include in the analysis involved considering the following criteria. The trials must have been either nonrandomized comparative trials or randomized controlled trials (RCTs) that compared the effectiveness of Atez/Bev to that of lenvatinib in treating advanced HCC patients. Additionally, the study sample size had to consist of at least 50 patients and the clinical data, such as OS, PFS or treatment response, had to be reported. Only studies published in English were considered. On the other hand, studies that did not compare Atez/Bev with lenvatinib, did not report efficacy or safety data, reported data for fewer than 50 patients, or were conference abstracts, case reports, reviews, study protocols and editorials were excluded from the study.

### 2.3. Quality assessment and data extraction

The Newcastle Ottawa Scale were used to assess the quality of included studies because most of them were retrospective. Two independent authors reviewed and scored each article and then discussed it until their results were consistent. Studies with 4–6 and 7–9 validity scores were regarded to be of low and high quality, respectively. Then 2 independent reviewers extracted the data from the eligible studies: author names, year of publication, study design, sample size, baseline characteristics of the study population, treatment regimens, primary outcome measures, and adverse events.

### 2.4. Definition of outcomes

In the study, the primary outcome was the OS, which was determined as the interval from treatment initiation to death or censorship. In addition to OS, secondary outcomes such as the incidence of adverse events (AE), ORR, PFS, and disease control rate (DCR) were also evaluated. To compute PFS, the duration from the start of treatment to tumor progression was established using radiological evidence. Tumor response assessment was conducted using the modified Response Evaluation Criteria in Solid Tumors methodology. ORR was determined as the combination of partial and complete responses, and DCR was calculated by adding stable disease, partial response, and complete response. AE were determined using the National Cancer Institute Common Terminology Criteria for Adverse Events (version 4.0).

### 2.5. Statistical analysis

Odds ratio (OR) with 95% confidence intervals (CIs) were reported as result of dichotomous data. The data were subjected to analysis using either a fixed-effects model or a random-effects model, depending on the level of heterogeneity observed. If the *I*^2^ statistic was greater than 50%, we considered the data to be heterogeneous. In such cases, we performed the random-effect model otherwise a fixed-effect model will be used. Review Manager 5.3 was used to perform all statistical analyses, *P* < .05 was considered statistically significant.

## 3. Results

**Figure 1.**
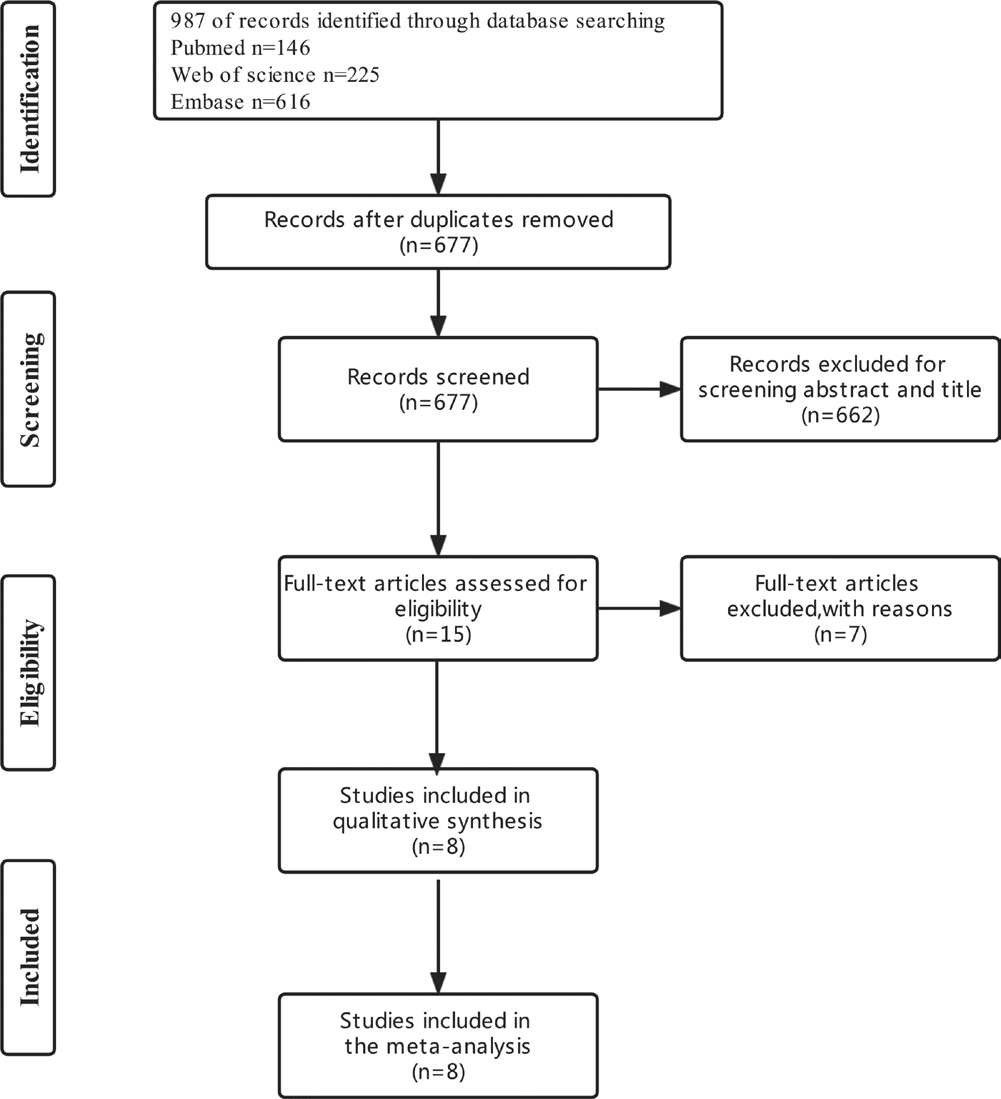
PRISMA flowchart.

Our search strategy led to the identification of 987 studies from Pubmed, EMBASE, and Web of Science databases. After eliminating 310 duplicate studies, we examined the abstracts and titles of the remaining articles and obtained the full text of 15 of them. A thorough review resulted in the inclusion of 8 studies that fully satisfied the criteria (Fig. 1).

All 8 studies that were analyzed were retrospective and considered to be of high quality (Tables 1 and 2). Over 7 years from 2015 to 2022, 6628 patients diagnosed with advanced liver cancer (HCC) received treatment with either the combination of Atez/Bev (n = 2492) or lenvatinib (n = 4136). Three of the studies were performed across multiple centers, while three were conducted in Japan, one in China, and one in Korea.

**Table 1:** Characteristics of included studies.

Study	Recruitment year	Country	Design	Group	Cases	Age (yr)	Sex (M/F)	Etiology (viral/non-viral)	ECOG PS (0/>0)	Child–Pugh (A/B)	BCLC stage (A&B/C)	Macrovascular invasion	Extrahepatic metastasis
Andrea Casadei-Gardini 2022	2015–2022	Multicenter	RCS	Atez/Bev	864	72 (IQR 64, 78)	682/182	484/380	679/185	778/86	NA	185 (21.5%)	285 (21.2%)
				Lenvatinib	1343	72 (IQR 65, 79)	1058/285	758/585	1057/286	1211/132	NA	316 (36.6%)	491 (36.6%)
Kazuki Maesaka 2022	2018–2021	Japan	PCS	Atez/Bev	66	76 (49–93)	50/16	36/30	60/6	64/2	31/35	12 (18.2%)	27 (40.9%)
				Lenvatinib	66	73 (53–91)	48/18	37/29	56/10	62/4	40/26	11 (16.7%)	18 (27.3%)
Beom Kyung Kim 2022	2019–2021	Korea	RCS	Atez/Bev	86	62 (56–71)	70/16	65/21	36/50	82/4	18/68	43 (50.0%)	37 (43.0%)
				Lenvatinib	146	62 (55–70)	124/22	109/37	105/41	127/19	14/132	76 (52.1%)	91 (62.3%)
Chung-Wei Su 2022	2018–2022	China	RCS	Atez/Bev	46	61.2 (38.4–83.9)	38/8	41/5	18/28	40/6	14/32	24 (52.2%)	15 (32.7%)
				Lenvatinib	46	69.6 (39.8–86.9)	38/8	38/8	24/22	41/5	16/30	24 (52.2%)	17 (37.0%)
Atsushi Hiraoka 2022	2020–2022	Japan	RCS	Atez/Bev	194	74 (68–79)	148/46	102/92	167/27	194/0	93/101	44 (22.7%)	71 (36.6%)
				Lenvatinib	57	73 (69–79)	41/16	27/30	47/10	57/0	34/23	5 (8.8%)	15 (26.3%)
Takashi Niizeki 2022	2018–2022	Japan	RCS	Atez/Bev	161	73 (38–93)	123/38	85/76	NA	NA	87/74	35 (21.7%)	47 (29.2)
				Lenvatinib	568	72 (31–93)	467/101	318/250	NA	NA	293/275	106 (18.7%)	204 (35.9%)
M. Rimini 2022	2017–2022	Multicenter	PCS	Atez/Bev	190	<75/≥75 (111/79)	149/41	NA	142/48	179/11	85/105	46 (24.2%)	NA
				Lenvatinib	569	<75/≥75 (319/250)	457/112	NA	466/103	488/81	235/334	462 (81.2)	NA
Mara Persano 2022	2010–2022	Multicenter	RCS	Atez/Bev	885	≤70/>70 (339/484)	657/228	442/381	615/208	769/54	335/488	175 (21.3%)	305 (37.1%)
				Lenvatinib	1341	≤70/>70 (598/714)	1032/309	763/549	1088/224	1166/146	554/758	260 (19.8%)	477 (36.4%)

Atez/Bev = atezolizumab plus bevacizumab, IQR = inter quartile range, NA = not available, PCS = prospective cohort study, RCS = retrospective control study.

**Table 2:** Risk of bias for inclusion studies.

Study, year	Selection	Comparability	Outcome	NOS score
Andrea Casadei-Gardini, 2022	4	1	3	8
Kazuki Maesaka, 2022	3	2	2	7
Beom Kyung Kim, 2022	3	1	3	7
Chung-Wei Su, 2022	3	2	3	8
Atsushi Hiraoka, 2022	4	1	3	8
Takashi Niizeki, 2022	4	1	2	7
M. Rimini, 2022	3	2	2	7
Mara Persano, 2022	4	1	3	8

NOS = Newcastle Ottawa Scale.

### 3.1. OS and PFS

**Figure 2.**
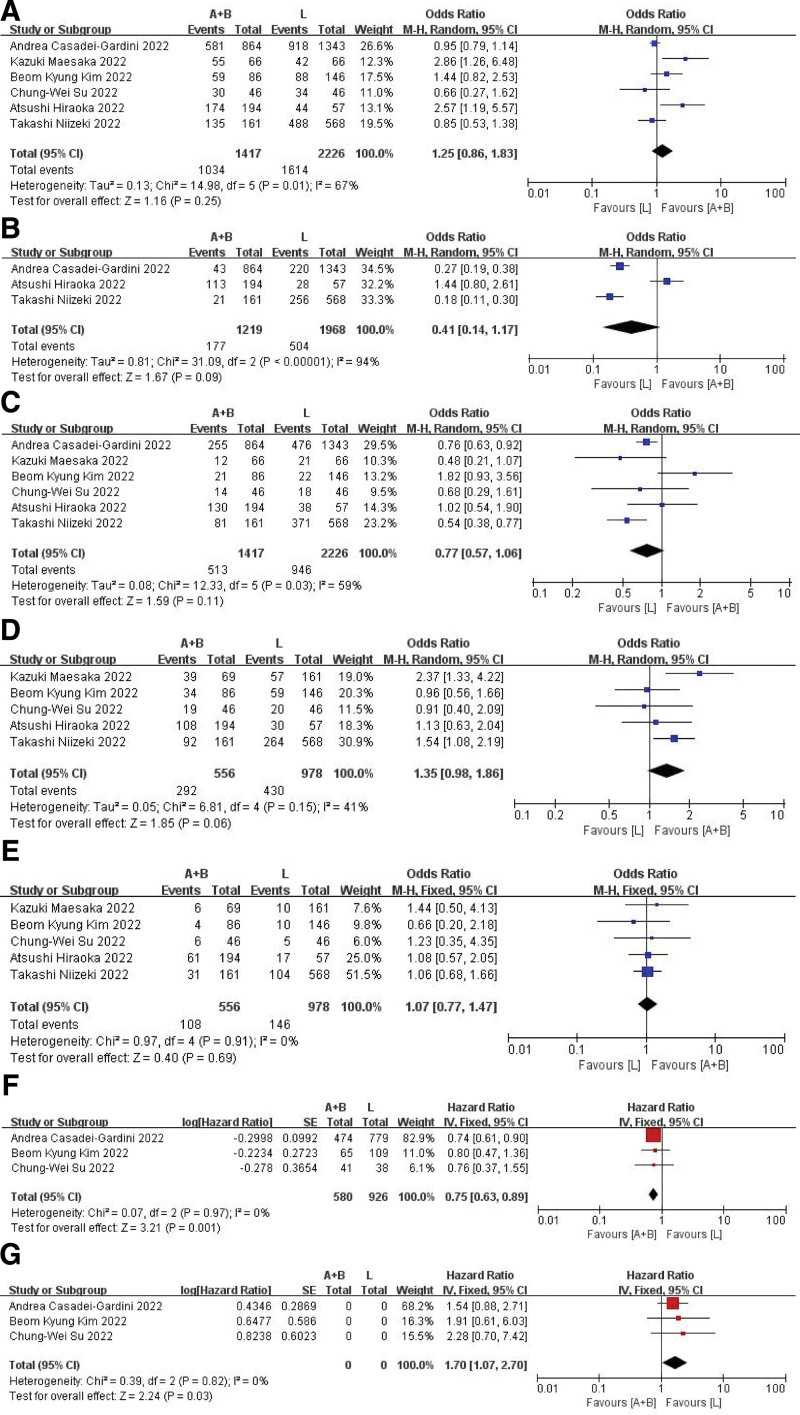
Forest plots for OS and PFS. Six-month (A), 12 mo (B), and 18 mo (C). OS forest plot; 6-mo (D) and 12 mo (E) PFS forest plot; (F) the mOS of viral-etiology group; (G) the mOS of Child–Pugh class B liver function group. OS = overall survival, PFS = progression-free survival.

As most of the studies in the review lacked complete data on OS and PFS, substitute metrics were utilized in the form of 0.5-, 1-, and 1.5-year OS or PFS rates. As shown in Figure 2A–E, there was no significant difference in 0.5-, 1-, 1.5-year OS rates and 0.5-, 1-year PFS rates between the 2 groups. At the same time, we performed subgroup analysis and indicated that patients with HCC caused by viral hepatitis would benefit more from the Atez/Bev therapy but patients with a Child–Pugh class B liver function would benefit more from Lenvatinib (Fig. 2F and G).

### 3.2. Treatment response

**Figure 3.**
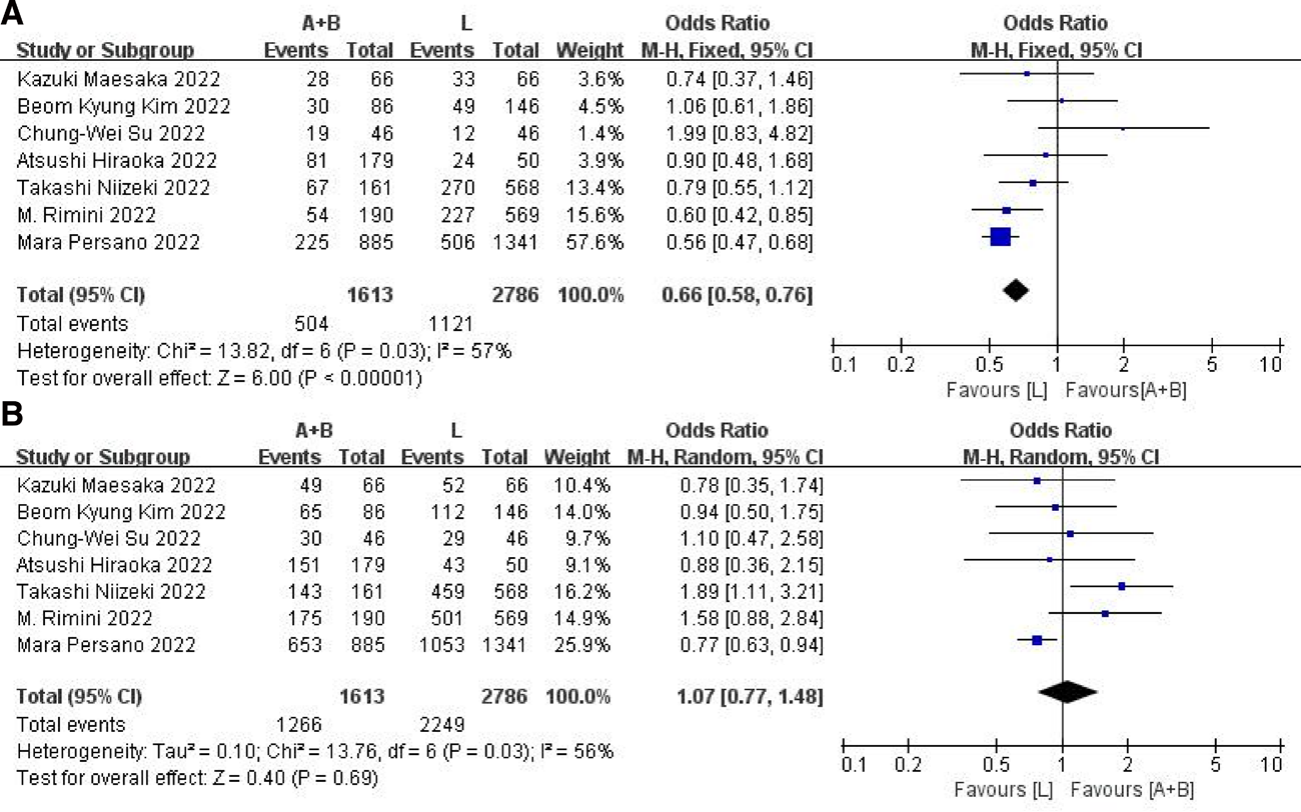
Objective response rate and disease control rate forest plots. (A) Objective response rate forest plot. (B) Disease control rate forest plot.

In 7 studies, both ORR and DCR were documented. Due to the high heterogeneity (*I*^2^ = 57%/56% in ORR/DCR) observed among the studies, a random-effect model was employed for further analysis. According to the findings, lenvatinib exhibited significantly greater ORR than Atez/Bev (OR: 0.76, 95% CI: 0.59–0.98, *P* = .004) (Fig. 3A). However, there was no significant difference in DCR between the 2 groups, with a combined OR of 1.07 (95% CI: 0.77–1.48, *P* = .069) (Fig. 3B).

### 3.3. Safety

**Figure 4.**
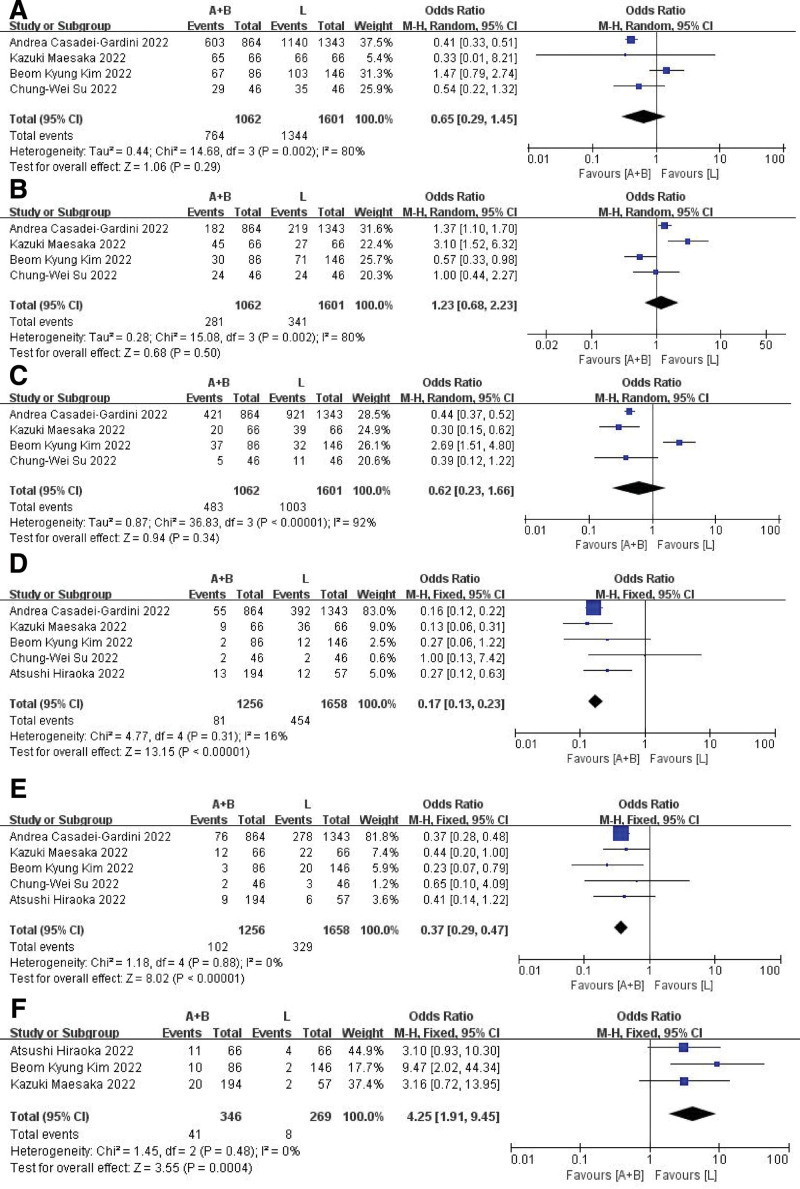
Adverse event forest plot. Overall (A), grades 1–2 (B), and grades 3–4 (C). AEs rate forest plot; incidence rate of hypothyroidism (D), diarrhea (E), and rash (F). AEs = adverse events.

Four studies (comprising 2663 evaluable patients) reported the overall incidence of AEs. Among patients treated with Atez/Bev and lenvatinib, a combined prevalence of 71.9% and 83.9%, respectively, was observed. Meanwhile, no significant difference in the overall, grade 1–2 and grade 3–4 AEs rate between the 2 groups was found in the random-effect model (Fig. 4A–C). Patients receiving lenvatinib treatment face a greater risk of experiencing hypothyroidism and diarrhea, whereas those treated with Atez/Bev are more prone to developing a rash (Fig. 4D–F).

## 4. Discussion

In the past decade, sorafenib was considered the only first-line systemic treatment for advanced unresectable HCC, based on the SHARP and Asia-Pacific trials.^[17,18]^ However, the REFLECT and IMbrave 150 trials demonstrated that lenvatinib and Atez/Bev were more effective than sorafenib in terms of higher ORR, better OS, and PFS.^[7,8]^ As a result, lenvatinib and Atez/Bev were recommended as the first-line drug for the treatment of advanced HCC. Unlike the inclusion criteria specified in the RCTs, the indications of these 2 treatments were expanded in the real world.

To this day, no meta-analysis has compared the effectiveness and safety of lenvatinib and Atez/Bev in the treatment of unresectable HCC under real-world conditions. Our meta-analysis showed that there was no significant difference in the 0.5-, 1-, and 1.5-year OS rates and 0.5-, 1-year PFS rates between the 2 treatments. Subgroup analysis indicated that Atez/Bev therapy was more beneficial for patients with HCC caused by viral hepatitis, while lenvatinib was more beneficial for patients with a Child–Pugh class B liver function. Additionally, lenvatinib had significantly higher ORR than Atez/Bev, but there was no significant difference in DCR between the 2 treatments. The overall incidence of AEs, as well as the rates of grade 1–2 and grade 3–4 AEs, did not differ significantly between the 2 treatments. However, patients receiving lenvatinib treatment had a higher risk of hypothyroidism and diarrhea, while those treated with Atez/Bev were more prone to developing a rash.

While there may not be any statistically significant differences, most research suggests that lenvatinib is more effective in terms of prolonging the OS and PFS of advanced HCC patients, as well as achieving a higher ORR. Based on earlier evidence, increased familiarity with managing sorafenib was associated with superior survival outcomes.^[19,20]^ Given that sorafenib and lenvatinib fall into the same category of drugs, with numerous pharmacological similarities, and that sorafenib has been utilized as a first-line treatment for more than 10 years in clinical practice, it’s reasonable to assume that physicians who have worked with sorafenib before may require less time to learn how to manage the AEs of lenvatinib. This might be the reason why clinical outcomes reported in real-world studies are better than those found in randomized trials.

On the contrary, in the real world, the effectiveness of Atez/Bev for treating HCC is worse than what the registered studies have shown. Atez/Bev is the first approved combination of immunotherapy used for HCC, which means that even many medical professionals who specialize in treating HCC are encountering this type of treatment for the first time. Thus, even though immunotherapy has better safety and is more manageable than TKIs, it is crucial to consider that it takes time to learn how to manage new therapies. Currently, there is limited information comparing the efficacy of Atez/Bev to that of lenvatinib, and more RCT will be required in the future to confirm the results.

Our analysis has shown that Atez/Bev provides a significant advantage in terms of OS for patients with a viral etiology. Furthermore, previous studies suggest that individuals with HCC who have non-viral etiologies, such as nonalcoholic steatohepatitis/nonalcoholic fatty liver disease, are more likely to experience benefits from lenvatinib.^[16]^

This discovery aligns with recent evidence that emphasizes the role of etiology in advanced HCC, especially in patients receiving treatment with only anti-programmed death ligand-1 or with anti-vascular endothelial growth factor. In contrast, the HIMALAYA trial demonstrated the effectiveness of an anti-programmed death ligand-1 plus anti-cytotoxic T lymphocyte antigen 4 in non-viral patients.^[21]^ It has been suggested that etiology (viral versus non-viral) plays a vital role in HCC biology and the host immune response, and that patients with nonalcoholic steatohepatitis/nonalcoholic fatty liver disease-related HCC may not respond as well to immunotherapy. Abou-Alfa et al^[22]^ discovered a connection between an increase in hepatic CD8^+^PD1^+^ T cells caused by immunotherapy and the impairment of immune surveillance, leading to hepatocarcinogenesis in a mouse model of nonalcoholic steatohepatitis. In the same study, a meta-analysis was conducted on three phase 3 immunotherapy studies, which revealed no survival advantages from immunotherapy in patients with non-viral etiology. However, patients with HBV and HCV showed improved survival rates.^[23]^ To date, the findings can only be viewed as hypothesis-generating.

In clinical practice, these 2 treatment regimens are not mutually exclusive but can complement each other. Johira et al^[24]^ and Yano et al^[25]^ have both reported even if patients do not respond to Atez/Bev, it is possible for them to benefit from treatment lenvatinib. Some patients may regain the opportunity for curative surgery and achieve complete pathological remission.

This study has several potential limitations. Firstly, because there were no RCTs examining the effectiveness of Atez/Bev or Lenvatinib in the treatment of advanced HCC, a large number of NRCT studies were included in this meta-analysis, which may have resulted in selection bias. Secondly, significant heterogeneity was observed among some of the study outcomes, which could be due to a variety of factors such as the quality of the NRCT studies, the small number of studies included in subset analyses, and differences in patient characteristics. The limitations outlined above could have impacted the results of this meta-analysis.

## 5. Conclusion

To conclude, our study did not find any significant difference in effectiveness and safety between Atez/Bev and Lenvatinib. Nonetheless, there are indications that lenvatinib treatment may be more beneficial for patients with Child–Pugh class B liver function, and Atezolizumab plus Bevacizumab may be more effective for those with viral etiology. However, larger prospective studies are necessary to validate these findings.

## Author contributions

**Conceptualization:** Sihao Du, Ke Cao, Dongdong Lin.

**Data curation:** Sihao Du, Ke Cao.

**Formal analysis:** Sihao Du, Ke Cao, Dongdong Lin.

**Funding acquisition:** Zhenshun Wang.

**Investigation:** Ke Cao, Dongdong Lin.

**Methodology:** Sihao Du, Ke Cao.

**Project administration:** Zhenshun Wang, Dongdong Lin.

**Resources:** Sihao Du, Ke Cao.

**Software:** Sihao Du, Ke Cao.

**Supervision:** Sihao Du, Zhenshun Wang, Dongdong Lin.

**Validation:** Zhenshun Wang.

**Visualization:** Sihao Du.

**Writing – original draft:** Sihao Du, Ke Cao.

**Writing – review & editing:** Zhenshun Wang, Dongdong Lin.

## Supplementary Material




