# Supplementary material for: Clinical efficacy and safety of atezolizumab plus bevacizumab versus lenvatinib in the treatment of advanced hepatocellular carcinoma: A systematic review and meta-analysis
Source: Medicine (Baltimore). 2023 Jun 9;102(23):e33852. doi: 10.1097/MD.0000000000033852 (PMC10256357; doi:10.1097/MD.0000000000033852)
Supplement: Supplementary file 2 [file medi-102-e33852-s002.pdf]

Supplementary file 1: Our search strategy in pubmed.

((((((((((((((((((((Carcinomas, Hepatocellular) OR (Hepatocellular Carcinomas)) OR (Liver Cell Carcinoma, Adult)) OR (Liver Cancer, Adult)) OR (Adult Liver Cancer)) OR (Adult Liver Cancers)) OR (Cancer, Adult Liver)) OR (Cancers, Adult Liver)) OR (Liver Cancers, Adult)) OR (Liver Cell Carcinoma)) OR (Carcinoma, Liver Cell)) OR (Carcinomas, Liver Cell)) OR (Cell Carcinoma, Liver)) OR (Cell Carcinomas, Liver)) OR (Liver Cell Carcinomas)) OR (Hepatocellular Carcinoma)) OR (Hepatoma)) OR (Hepatomas)) AND ((((((((Atezolizumab) OR (anti-PDL1)) OR (immunoglobulin G1, anti-(human CD antigen CD274) (human monoclonal MDPL3280a heavy chain), disulfide with human monoclonal MDPL3280a kappa-chain, dimer)) OR (MPDL3280A)) OR (MPDL-3280A)) OR (Tecentriq)) OR (RG7446)) OR (RG-7446))) AND (((((Bevacizumab) OR (Mvasi)) OR (Bevacizumab-awwb)) OR (Bevacizumab awwb)) OR (Avastin))) AND ((((((((((((((((Lenvatinib) OR (4-(3-chloro-4-(N'-cyclopropylureido)phenoxy)-7-methoxyquinoline-6-carboxamide)) OR (4-(3-chloro-4-((cyclopropylaminocarbonyl)amino)phenoxy)-7-methoxy-6-quinolinecarboxamide)) OR (Lenvima)) OR (E 7080)) OR (E-7080)) OR (ER-203492-00)) OR (E7080)) OR (E-7080 mesylate)) OR (E7080 mesylate)) OR (lenvatinib metabolite M2)) OR (4-(3-chloro-4-(((cyclopropylamino)carbonyl)amino)phenoxy)-7-hydroxy-6-quinolinecarboxamide)) OR (lenvatinib mesylate)) OR (lenvatinib methanesulfonate)) OR (N-(4-((6-carbamoyl-7-methoxyquinolin-4-yl)oxy)-2-chlorophenyl)-N'-cyclopropylurea monomethanesulfonate)) OR (lenvatinib mesilate))

Translations

Carcinomas, Hepatocellular: "carcinoma, hepatocellular"[MeSH Terms] OR ("carcinoma"[All Fields] AND "hepatocellular"[All Fields]) OR "hepatocellular carcinoma"[All Fields] OR ("carcinomas"[All Fields] AND "hepatocellular"[All Fields]) OR "carcinomas, hepatocellular"[All Fields]

Hepatocellular Carcinomas: "carcinoma, hepatocellular"[MeSH Terms] OR ("carcinoma"[All Fields] AND "hepatocellular"[All Fields]) OR "hepatocellular carcinoma"[All Fields] OR ("hepatocellular"[All Fields] AND "carcinomas"[All Fields]) OR "hepatocellular carcinomas"[All Fields]

Liver Cell Carcinoma, Adult: "carcinoma, hepatocellular"[MeSH Terms] OR ("carcinoma"[All Fields] AND "hepatocellular"[All Fields]) OR "hepatocellular carcinoma"[All Fields] OR ("liver"[All Fields] AND "cell"[All Fields] AND "carcinoma"[All Fields] AND "adult"[All Fields]) OR "liver cell carcinoma, adult"[All Fields]

Liver Cancer, Adult: "carcinoma, hepatocellular"[MeSH Terms] OR ("carcinoma"[All Fields] AND "hepatocellular"[All Fields]) OR "hepatocellular carcinoma"[All Fields] OR ("liver"[All Fields] AND "cancer"[All Fields] AND "adult"[All Fields]) OR "liver cancer, adult"[All Fields]

Adult Liver Cancer: "carcinoma, hepatocellular"[MeSH Terms] OR ("carcinoma"[All Fields] AND "hepatocellular"[All Fields]) OR "hepatocellular carcinoma"[All Fields] OR ("adult"[All Fields] AND "liver"[All Fields] AND "cancer"[All Fields]) OR "adult liver cancer"[All Fields]

Adult Liver Cancers: "carcinoma, hepatocellular"[MeSH Terms] OR ("carcinoma"[All Fields] AND "hepatocellular"[All Fields]) OR "hepatocellular carcinoma"[All Fields] OR ("adult"[All Fields] AND "liver"[All Fields] AND "cancers"[All Fields]) OR "adult liver cancers"[All Fields]

Cancer, Adult Liver: "carcinoma, hepatocellular"[MeSH Terms] OR ("carcinoma"[All Fields] AND "hepatocellular"[All Fields]) OR "hepatocellular carcinoma"[All Fields] OR ("cancer"[All Fields] AND "adult"[All Fields] AND "liver"[All Fields]) OR "cancer, adult liver"[All Fields]

Cancers, Adult Liver: "carcinoma, hepatocellular"[MeSH Terms] OR ("carcinoma"[All Fields] AND "hepatocellular"[All Fields]) OR "hepatocellular carcinoma"[All Fields] OR ("cancers"[All Fields] AND "adult"[All Fields] AND "liver"[All Fields]) OR "cancers, adult liver"[All Fields]
Liver Cancers, Adult: "carcinoma, hepatocellular"[MeSH Terms] OR ("carcinoma"[All Fields] AND "hepatocellular"[All Fields]) OR "hepatocellular carcinoma"[All Fields] OR ("liver"[All Fields] AND "cancers"[All Fields] AND "adult"[All Fields]) OR "liver cancers, adult"[All Fields]
Liver Cell Carcinoma: "carcinoma, hepatocellular"[MeSH Terms] OR ("carcinoma"[All Fields] AND "hepatocellular"[All Fields]) OR "hepatocellular carcinoma"[All Fields] OR ("liver"[All Fields] AND "cell"[All Fields] AND "carcinoma"[All Fields]) OR "liver cell carcinoma"[All Fields]
Carcinoma, Liver Cell: "carcinoma, hepatocellular"[MeSH Terms] OR ("carcinoma"[All Fields] AND "hepatocellular"[All Fields]) OR "hepatocellular carcinoma"[All Fields] OR ("carcinoma"[All Fields] AND "liver"[All Fields] AND "cell"[All Fields]) OR "carcinoma, liver cell"[All Fields]
Carcinomas, Liver Cell: "carcinoma, hepatocellular"[MeSH Terms] OR ("carcinoma"[All Fields] AND "hepatocellular"[All Fields]) OR "hepatocellular carcinoma"[All Fields] OR ("carcinomas"[All Fields] AND "liver"[All Fields] AND "cell"[All Fields]) OR "carcinomas, liver cell"[All Fields]
Cell Carcinoma, Liver: "carcinoma, hepatocellular"[MeSH Terms] OR ("carcinoma"[All Fields] AND "hepatocellular"[All Fields]) OR "hepatocellular carcinoma"[All Fields] OR ("cell"[All Fields] AND "carcinoma"[All Fields] AND "liver"[All Fields]) OR "cell carcinoma, liver"[All Fields]
Cell Carcinomas, Liver: "carcinoma, hepatocellular"[MeSH Terms] OR ("carcinoma"[All Fields] AND "hepatocellular"[All Fields]) OR "hepatocellular carcinoma"[All Fields] OR ("cell"[All Fields] AND "carcinomas"[All Fields] AND "liver"[All Fields]) OR "cell carcinomas, liver"[All Fields]
Liver Cell Carcinomas: "carcinoma, hepatocellular"[MeSH Terms] OR ("carcinoma"[All Fields] AND "hepatocellular"[All Fields]) OR "hepatocellular carcinoma"[All Fields] OR ("liver"[All Fields] AND "cell"[All Fields] AND "carcinomas"[All Fields]) OR "liver cell carcinomas"[All Fields]
Hepatocellular Carcinoma: "carcinoma, hepatocellular"[MeSH Terms] OR ("carcinoma"[All Fields] AND "hepatocellular"[All Fields]) OR "hepatocellular carcinoma"[All Fields] OR ("hepatocellular"[All Fields] AND "carcinoma"[All Fields])
Hepatoma: "carcinoma, hepatocellular"[MeSH Terms] OR ("carcinoma"[All Fields] AND "hepatocellular"[All Fields]) OR "hepatocellular carcinoma"[All Fields] OR "hepatoma"[All Fields] OR "hepatomas"[All Fields]
Hepatomas: "carcinoma, hepatocellular"[MeSH Terms] OR ("carcinoma"[All Fields] AND "hepatocellular"[All Fields]) OR "hepatocellular carcinoma"[All Fields] OR "hepatoma"[All Fields] OR "hepatomas"[All Fields]
Atezolizumab: "atezolizumab"[Supplementary Concept] OR "atezolizumab"[All Fields]
anti-PDL1: "atezolizumab"[Supplementary Concept] OR "atezolizumab"[All Fields] OR "anti pdl1"[All Fields]
immunoglobulin G1,: "immunoglobulin g"[MeSH Terms] OR "immunoglobulin g"[All Fields] OR ("immunoglobulin"[All Fields] AND "g1"[All Fields]) OR "immunoglobulin g1"[All Fields]
anti-: "Antib Technol J"[Journal:__jid9918350789006676] OR "anti"[All Fields]
human: "human's"[All Fields] OR "humans"[MeSH Terms] OR "humans"[All Fields] OR "human"[All Fields]
CD antigen: "antigens, cd"[MeSH Terms] OR ("antigens"[All Fields] AND "cd"[All Fields]) OR "cd antigens"[All Fields] OR ("cd"[All Fields] AND "antigen"[All Fields]) OR "cd antigen"[All Fields]

human: "human's"[All Fields] OR "humans"[MeSH Terms] OR "humans"[All Fields] OR "human"[All Fields]
monoclonal: "monoclonal"[All Fields] OR "monoclonality"[All Fields] OR "monoclonally"[All Fields] OR "monoclonals"[All Fields] OR "monoclone"[All Fields] OR "monoclones"[All Fields]
chain: "chain"[All Fields] OR "chain's"[All Fields] OR "chains"[All Fields]
, disulfide: "disulfides"[MeSH Terms] OR "disulfides"[All Fields] OR "disulfide"[All Fields] OR "disulphide"[All Fields] OR "disulphides"[All Fields]
human: "human's"[All Fields] OR "humans"[MeSH Terms] OR "humans"[All Fields] OR "human"[All Fields]
monoclonal: "monoclonal"[All Fields] OR "monoclonality"[All Fields] OR "monoclonally"[All Fields] OR "monoclonals"[All Fields] OR "monoclone"[All Fields] OR "monoclones"[All Fields]
dimer: "dimer"[All Fields] OR "dimer's"[All Fields] OR "dimeric"[All Fields] OR "dimerisation"[All Fields] OR "dimerise"[All Fields] OR "dimerised"[All Fields] OR "dimerises"[All Fields] OR "dimerising"[All Fields] OR "dimerization"[MeSH Terms] OR "dimerization"[All Fields] OR "dimerizations"[All Fields] OR "dimerize"[All Fields] OR "dimerized"[All Fields] OR "dimerizer"[All Fields] OR "dimerizers"[All Fields] OR "dimerizes"[All Fields] OR "dimerizing"[All Fields] OR "dimers"[All Fields]
MPDL3280A: "atezolizumab"[Supplementary Concept] OR "atezolizumab"[All Fields] OR "mpdl3280a"[All Fields]
MPDL-3280A: "atezolizumab"[Supplementary Concept] OR "atezolizumab"[All Fields] OR "mpdl 3280a"[All Fields]
Tecentriq: "atezolizumab"[Supplementary Concept] OR "atezolizumab"[All Fields] OR "tecentriq"[All Fields]
RG7446: "atezolizumab"[Supplementary Concept] OR "atezolizumab"[All Fields] OR "rg7446"[All Fields]
RG-7446: "atezolizumab"[Supplementary Concept] OR "atezolizumab"[All Fields] OR "rg 7446"[All Fields]
Bevacizumab: "bevacizumab"[MeSH Terms] OR "bevacizumab"[All Fields] OR "bevacizumab's"[All Fields]
Mvasi: "bevacizumab"[MeSH Terms] OR "bevacizumab"[All Fields] OR "mvasi"[All Fields] OR "bevacizumab's"[All Fields]
Bevacizumab-awwb: "bevacizumab"[MeSH Terms] OR "bevacizumab"[All Fields] OR ("bevacizumab"[All Fields] AND "awwb"[All Fields]) OR "bevacizumab awwb"[All Fields]
Bevacizumab awwb: "bevacizumab"[MeSH Terms] OR "bevacizumab"[All Fields] OR ("bevacizumab"[All Fields] AND "awwb"[All Fields]) OR "bevacizumab awwb"[All Fields]
Avastin: "bevacizumab"[MeSH Terms] OR "bevacizumab"[All Fields] OR "avastin"[All Fields] OR "bevacizumab's"[All Fields]
Lenvatinib: "lenvatinib"[Supplementary Concept] OR "lenvatinib"[All Fields]
phenoxy: "phenoxy"[All Fields] OR "phenoxys"[All Fields]
amino: "amino"[All Fields] OR "aminos"[All Fields]
phenoxy: "phenoxy"[All Fields] OR "phenoxys"[All Fields]
Lenvima: "lenvatinib"[Supplementary Concept] OR "lenvatinib"[All Fields] OR "lenvima"[All Fields]
E 7080: "lenvatinib"[Supplementary Concept] OR "lenvatinib"[All Fields] OR "e 7080"[All Fields]
E-7080: "lenvatinib"[Supplementary Concept] OR "lenvatinib"[All Fields] OR "e 7080"[All Fields]

ER-203492-00: "lenvatinib"[Supplementary Concept] OR "lenvatinib"[All Fields] OR "er 203492 00"[All Fields]
E7080: "lenvatinib"[Supplementary Concept] OR "lenvatinib"[All Fields] OR "e7080"[All Fields]
E-7080 mesylate: "lenvatinib"[Supplementary Concept] OR "lenvatinib"[All Fields] OR "e 7080 mesylate"[All Fields]
E7080 mesylate: "lenvatinib"[Supplementary Concept] OR "lenvatinib"[All Fields] OR "e7080 mesylate"[All Fields]
lenvatinib metabolite M2: "lenvatinib"[Supplementary Concept] OR "lenvatinib"[All Fields] OR "lenvatinib metabolite m2"[All Fields]
carbonyl: "carbonyl"[All Fields] OR "carbonylated"[All Fields] OR "carbonylating"[All Fields] OR "carbonylation"[All Fields] OR "carbonylations"[All Fields] OR "carbonylative"[All Fields] OR "carbonylic"[All Fields] OR "carbonyls"[All Fields]
amino: "amino"[All Fields] OR "aminos"[All Fields]
phenoxy: "phenoxy"[All Fields] OR "phenoxys"[All Fields]
lenvatinib mesylate: "lenvatinib"[Supplementary Concept] OR "lenvatinib"[All Fields] OR "lenvatinib mesylate"[All Fields]
lenvatinib methanesulfonate: "lenvatinib"[Supplementary Concept] OR "lenvatinib"[All Fields] OR "lenvatinib methanesulfonate"[All Fields]
lenvatinib mesilate: "lenvatinib"[Supplementary Concept] OR "lenvatinib"[All Fields] OR "lenvatinib mesilate"[All Fields]
